# Association between pre-stroke frailty status and stroke risk and impact on outcomes: a systematic review and meta-analysis of 1,660,328 participants

**DOI:** 10.1007/s40520-024-02845-0

**Published:** 2024-09-11

**Authors:** Shu-Fan Chen, Hai-Han Li, Zi-Ning Guo, Ke-Yu Ling, Xiao-Li Yu, Fei Liu, Xiao-Ping Zhu, Xiaoping Zhu

**Affiliations:** 1 Department of Nursing, Shanghai Tenth People’s Hospital, Tongji University School of Medicine, No. 301, Yanchang Road, Jing ‘an District, Shanghai, China; 2 School of Nursing, Soochow University, Suzhou, China

**Keywords:** Frailty, Stroke risk, Mortality, mRs, Systematic review, Meta-analysis

## Abstract

The prevalence of frailty is increasing, and it is associated with increased risk of diseases and adverse outcomes. Although substantial research has focused on post-stroke frailty, understanding of pre-stroke frailty remains limited. Our aim was to synthesize literature on pre-stroke frailty and stroke risk to explore their relationship and impact on prognosis. A systematic search of multiple databases was conducted to identify cohort studies published until October 28, 2023. Meta-analysis was conducted using a random effects model. Heterogeneity was assessed with the I² statistic, and publication bias was evaluated using Begg’s test. Finally, we included 11 studies (*n* = 1,660,328 participants). The pooled hazard ratios (HRs) for stroke risk associated with pre-stroke frailty compared to non-frail individuals was 1.72 (95% confidence interval, CI: 1.46–2.02, *p* = 0.002, *I*^*2*^ = 69.2%, Begg’s test: *p* = 0.536). The pooled HRs for mortality and the pooled relative risk (RRs) modified Rankin Scale (mRs) associated with pre-stroke frailty were 1.68 (95% CI: 1.10–2.56, *p* = 0.136, *I*^*2*^ = 49.9%, Begg’s test: *p* = 0.296) and 3.11 (95% CI: 1.77–5.46, *p* = 0.192, *I*^*2*^ = 39.4%, Begg’s test: *p* = 1.000), respectively. In conclusion, pre-stroke frailty is strongly associated with stroke risk and impacts its prognosis, irrespective of the measurement method. Future research should focus on prospective studies to assess the effects of early intervention for frailty. This has significant implications for primary healthcare services and frailty management.

**Supplementary Information:**

The online version contains supplementary material available at 10.1007/s40520-024-02845-0.

## Introduction

Stroke is a group of acute cerebrovascular diseases (CVD) characterized by cerebral ischemia or hemorrhagic injury [1]. It is the second leading cause of death and disability in the world, affecting nearly 14 million people around the world every year and causing about 120 million disabilities. It not only reduces the quality of life of patients, but also increases the huge social health care burden [2–4]. Furthermore, patients with stroke may experience various complications, such as cognitive impairment, dysphagia, and malnutrition. Frailty, a complication of stroke, has garnered significant attention in recent years.

Age is a prominent risk indicator for common chronic diseases, frailty, and mortality. However, significant interindividual variation exists in the biological aging process and the rate of functional decline [5]. Frailty, as defined by the World Health Organization (WHO) and the ADVANTAGE Joint Action of the European Union, refers to a gradual age-related deterioration in physiological systems leading to diminished reserves of intrinsic capacity. This condition renders individuals highly susceptible to stressors and elevates the risk of various adverse health outcomes [6–8], making it a common geriatric syndrome and one of the biggest challenges facing the aging population [9]. Increasingly, geriatricians define frailty as a biological syndrome characterized by reduced reserve and resistance to stressors due to cumulative declines across various physiological systems, leading to vulnerability to adverse outcomes [10]. There is an increased risk of dependence and death when exposed to stressors like an acute stroke [6, 11]. Frailty is currently classified into two stages: pre-frailty and frailty [12]. Research indicates that frailty is prevalent in cases of acute stroke. At least one in four individuals who experience a stroke are found to be living with frailty, a figure that rises to two in three when pre-frailty is also taken into account [10]. Longitudinal evidence suggests that frailty increases overall cardiovascular disease incidence compared with adults without frailty [9, 13]. The incidence rates of pre-frailty and frailty before stroke were 49% and 22%, respectively [14]. Furthermore, research has shown that pre-stroke frailty is associated with adverse outcomes including mortality, disability, extended hospitalization, and impaired recovery post-illness [15]. Current evidence suggests that frailty may be reversible or delayed and, therefore, it is considered a public health problem [16]. Numerous healthcare systems worldwide are also integrating frailty measures into their acute care pathways [15]. Being able to identify which stroke patients are at the highest risk for poor functioning and mortality is critical given the need to weigh the risks, costs, and benefits of interventions in various shared decision-making processes [2].

Numerous studies have investigated the association of frailty after stroke with mortality and functional recovery after stroke [17–19]. There are also studies investigating the incidence of pre-stroke frailty, and some researchers have conducted relevant meta-analyses on the incidence of pre-stroke frailty. However, research on the influence of pre-stroke frailty on stroke remains in its early exploratory phase. The prevalence and effects of pre-stroke frailty on stroke risk and outcomes are not yet fully comprehended. Several studies [8, 20, 21] indicate that pre-stroke frailty correlates with stroke risk, although the strength of this association varies among studies. Additionally, the impact of pre-stroke frailty on other outcomes in stroke patients, such as modified Rankin Scale (mRs) and mortality, has not been clearly elucidated.

In order to offer a comprehensive and up-to-date overview of the association between pre-stroke frailty and stroke, we conducted a systematic review and meta-analysis of cohort studies. Our aim was to evaluate the relationship between pre-stroke frailty and stroke risk and prognosis, to clarify the need for enhanced screening and prevention of pre-stroke frailty. This approach could help reduce stroke incidence, improve patients’ quality of life, and promote healthy aging.

## Methods

This systematic review and meta-analysis is reported according to the PRISMA (Preferred Reporting Items for Systematic Reviews and Meta-Analyses) 2020 guideline [22]. The protocol was registered on the international prospective register of systematic reviews (PROSPERO; #CRD42023477942). The studies included in our review differ from those specified in the protocol. The protocol initially planned to include only prospective cohort studies. After a thorough literature search and review, we decided to include several relevant retrospective cohort studies to ensure a more comprehensive analysis.

### Data sources and search strategy

The search was conducted by two authors (CSF and LHH) using PubMed, Web of Science, Embase, Cochrane Library, CINAHL, PsycINFO, CNKI and Vip databases from their inception to October 28, 2023, using the search strategy shown in Table S1. We searched for cohort studies reporting on the association between pre-stroke frailty status and the risk or outcomes from the stroke. The search was limited to studies published in English or Chinese. Screening of titles and abstracts, as well as subsequent full-text reviews of potentially eligible studies, were performed independently by the two reviewers. Additionally, the reference lists of included studies were screened to identify additional relevant studies. Any disagreement and discordance between the two reviewers were resolved by the third reviewer (ZXP).

### Study eligibility criteria

The review question adhered to the PECO(S) framework as recommended [23]. Of interest were presumable healthy individuals in the general population or stroke patients, with no restrictions regarding age, sex (P), who exhibited frailty, with cutoff values for pre-frailty and frailty determined from original studies based on the usual practice or the authors’ classification (E), and compared them to those in a robust state (C), studies that reported relative risk (RR), hazard ratio (HR) and odds ratio (OR) with a 95% confidence interval (CI) between pre-stroke frailty status and risk or outcomes from stroke(O), and we focused entirely on reports from cohort studies (S).

We applied the following exclusion criteria: (a) animal and cell studies; (b) not relevant study design (e.g., intervention studies, cross-sectional studies, meta-analyses, reviews, and commentaries); (c) conference abstract; (d) the full literature is not available.

### Data extraction

Two reviewers (CSF and LHH) independently conducted the article screening, data extraction, and quality assessment. Extracted data included study characteristics like the primary author’s surname, publication year, region, average age, participant count, follow-up period, and outcomes like stroke risk, mortality, and mRS.

### Risk of bias assessment

We utilized the Newcastle-Ottawa Scale (NOS) to evaluate the risk of bias (ROB) in the studies included. This scale consists of 9 aspects for each study: 4 for selection, 2 for comparability, and 3 for outcome assessment. Each study could receive a maximum score of nine stars. Studies were categorized as low, moderate, or high quality based on their star ratings of 0–3, 4–6, and 7–9, respectively. As per the NOS scale: (a) One star was awarded if the exposure group accurately represented the average level of the community population. (b) One star was given for the selection of a non-exposed group from the same community as the exposed group. (c) One star was earned if exposure was identified through a reliable record or structured survey. (d) If no endpoint event occurred prior to the study commencement, one star was granted. (e) If study cohorts were comparable, such as through matching and controlling for confounding factors, one star was assigned. (f) study controls for any additional factor, one star was assigned. (g) Two stars were allocated if outcome events were assessed independently, blindly, or through data linkage. (h) Observation spanning over 5 years was rewarded with one star. (I) If all participants completed follow-up, one star was given. Two reviewers (CSF and LHH) independently assessed the quality of each included studies. Discrepancies were resolved through discussion, and if agreement could not be reached, a third author (ZXP) was consulted.

### Assessment of the quality of evidence

We used the Grading of Recommendations, Assessment, Development and Evaluation (GRADE) framework [24, 25] for cohort studies to assess the quality of evidence supporting the observed relation between pre-stroke frailty status and risk of stroke and its outcome we evaluated. Each estimate is rated as high, moderate, low, or very low quality [26] based on five domains: risk of bias [27], consistency [28], directness [29], precision [30] and publication bias [31]. Two reviewers (CSF and LHH) independently assessed the quality of each evidence. Discrepancies were resolved through discussion, and if agreement could not be reached, a third author (ZXP) was consulted. A complete description of the GRADE quality scoring is available in Table S4.

### Data analysis

RR and HR were used as the primary measures of association across multiple studies, and OR values were converted to RR values for further analysis. When both adjusted and unadjusted effect sizes were available, we prioritized adjusted ones. The synthesis of data was conducted using the random-effects model proposed by DerSimonian and Laird, which accommodates potential variability at the study level [32]. Statistical significance was determined by a two-tailed *p*-value < 0.05. To ensure robustness and reliability, meta-analytical results were visually presented through forest plots. Specifically, for studies encompassing both risk and outcomes of CVD, only data pertaining to stroke were included in the primary analysis.

Taking the logarithm of the RR and HR values prior to pooling them, and subsequently exponentiating the pooled results to derive the pooled RR and HR. Heterogeneity among studies was assessed utilizing Chi-squared-statistics [33] and *I*^*2*^-statistics [34], with the *p* value for Chi-squared statistics significant if < 0.05, and *I*^*2*^-percentage ranged from 0 to 100%. Subgroup analyses were conducted to explore potential sources of heterogeneity, stratified by follow-up period (≥ 5 vs. <5 years), frailty assessment tools (frailty phenotype, FP vs. frailty index, FI), study type (prospective cohort study vs. retrospective cohort study) and study quality (high vs. moderate).

Begg’ s test [35, 36]were used to investigate publication bias, and we considered a *p* value < 0.10 to indicate possible publication bias. To assess the reliability of the summary RRs and HRs and prevent them from being overly influenced by individual studies, we conducted sensitivity analyses. For each outcome, we recalculated the summary RRs and HRs using a “leave-one-out” methodology, systematically excluding each study one by one to evaluate its impact on the primary analysis. Statistical analyses were performed using Stata SE version 18 (StataCorp, TX, USA).

## Results

**Fig. 1:**
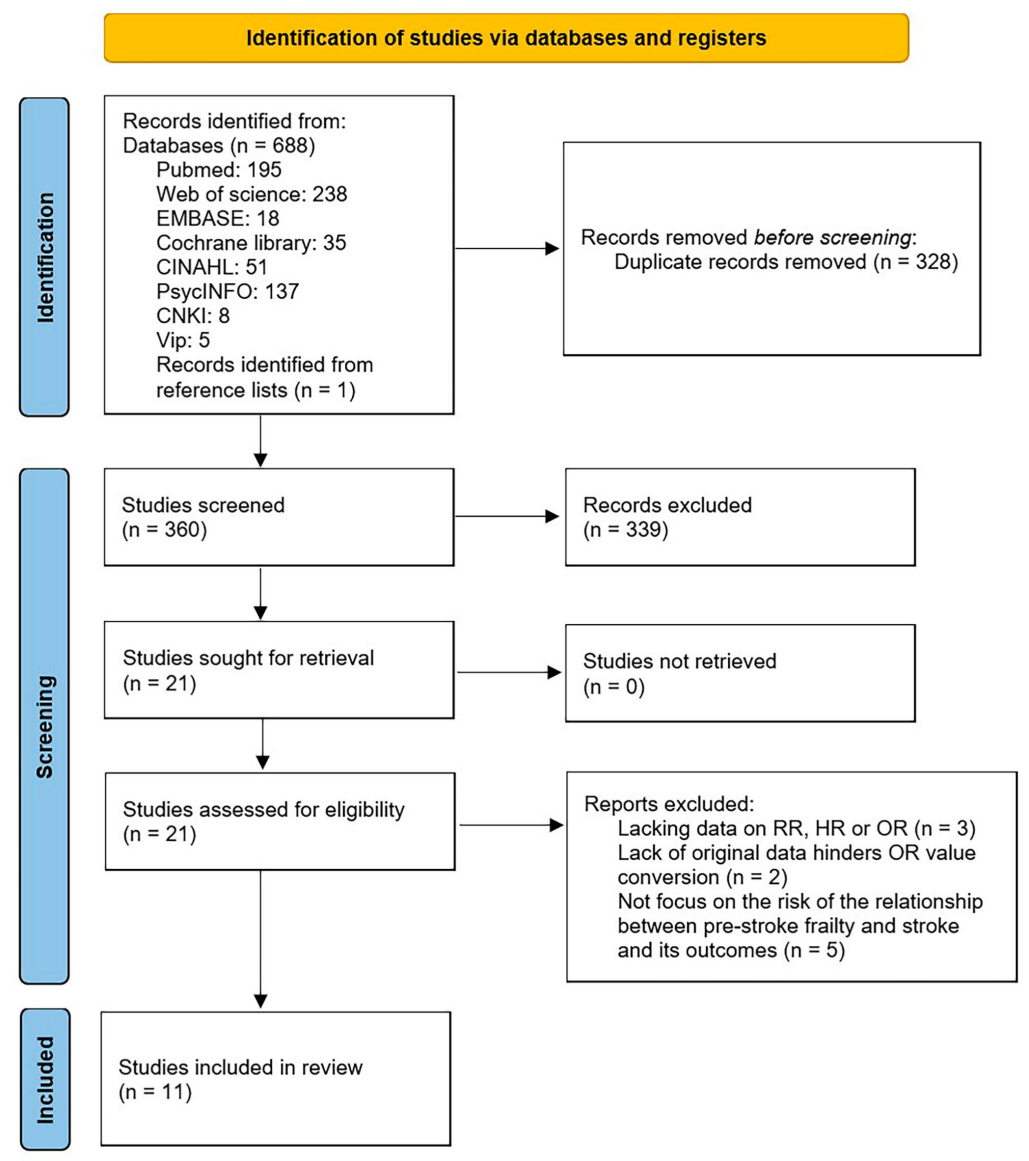
Flow diagram of the literature selection process

The process of literature search is depicted in Fig. 1. Following the removal of 328 duplicate citations, 360 unique articles were identified. Upon screening titles and abstracts, 339 citations were excluded, comprising 305 irrelevant studies, 10 meeting abstracts, 15 reviews, 5 meta-analyses, 2 cellular studies, and 2 laboratory studies. Subsequently, 21 studies underwent full-text examination. Among these, 10 citations were excluded for not meeting the inclusion criteria, such as lacking data on RR, HR, and OR, lacking original data necessary for OR conversion, or not focusing on the relationship between pre-stroke frailty and stroke outcomes. The studies excluded after the full-text eligibility assessment are detailed in Table S2. Ultimately, 11 independent cohort studies were deemed eligible for data synthesis.

### Study characteristics

Our search identified 688 citations in total, including 675 in English and 13 in Chinese. One study was identified by screening the reference lists of the included studies, while the others were retrieved through database searches. After removing duplicates and conducting title and abstract screening, as well as full-text assessment, we included 11 studies, encompassing a total of 1,660,328 individuals (Table 1). All eligible articles were published between 2013 and 2023. 9 studies were conducted in Asia; 1 in Europe; 1 in South America. 8 were prospective cohort studies; 3 were retrospective cohort studies; 6 were based on electronic medical records; and 5 were population-based studies. 3 studies enrolled patients with stroke, pre-stroke frailty status was obtained from recall or medical records. Regarding the frailty assessment tools used in the original studies, 5 studies utilized the FI proposed by Rockwood and Mitnitski, 1 employed the Simplified Frailty Index (SFI), 2 utilized the FP by Fried and colleagues, 1 utilized the FRAIL scale, and 2 studies used two frailty assessment tools: one used FI and PRISMA-7, the other used FI and FP. Based on the NOS, 7 studies were considered high-quality, while the rest were categorized as moderate-quality studies (Table S3).

**Table 1:** Characteristics of included studies regarding pre-stroke frailty and stroke risk and outcome

First author	Year	Geographic location	Study type	Frailty assessment	*N*		Age (years)	Outcome	Follow-up period
					M	F			
Jieun Jang [37]	2023	Asia	retrospective cohort	FI	451,833	517,052	N/A	stroke risk; mortality	>5 years
Tianqi Ma [38]	2023	Asia	prospective cohort	FP; FI	154,557	196,648	56.55	incidence of CMDs	>5 years
Liangkai Chen [9]	2023	Asia	prospective cohort	FP	146,152	167,941	55.90 (8.1)	CVD events	≤ 5 years
Chunxiu Wang [20]	2023	Asia	prospective cohort	FI	707	550	69.2 (8.0)	incidental CVD events; mortality	≤ 5 years
Fuxia Yang [39]	2022	Asia	prospective cohort	FRAIL	149	112	N/A	mRS	≤ 5 years
Luana Aparecida Miranda [2]	2022	South America	prospective cohort	PRISMA-7; FI	68	106	69	mortality; mRS	≤ 5 years
Xiao Liu [40]	2022	Asia	prospective cohort	FP	1747	3268	65.8 (7.6)	fatal CVD	>5 years
Xinyao Liu [41]	2022	Asia	retrospective cohort	FI	6787	6793	58.43 (9.54)	CVD events	>5 years
Rebecca Gugganig [17]	2021	Europe	prospective cohort	FI	1722	647	73 (8.0)	unplanned hospitalization	≤ 5 years
Madoka Noguchi [42]	2021	Asia	prospective cohort	SFI	145	87	76 (11)	mRS	≤ 5 years
Zhe Tang [43]	2013	Asia	retrospective cohort	FI	1593	1664	N/A	stroke risk	>5 years

Abbreviations: FI: the frailty index; FRAIL: the frail scale; PRISMA-7: the PRISMA-7 questionnaire; FP: the frailty phenotype; SFI: simplified frailty index; mRs: the modified Rankin Scale; CVD: cardiovascular disease; CMD: cardiometabolic diseases

### Pre-stroke frailty and stroke risk

**Fig. 2:**
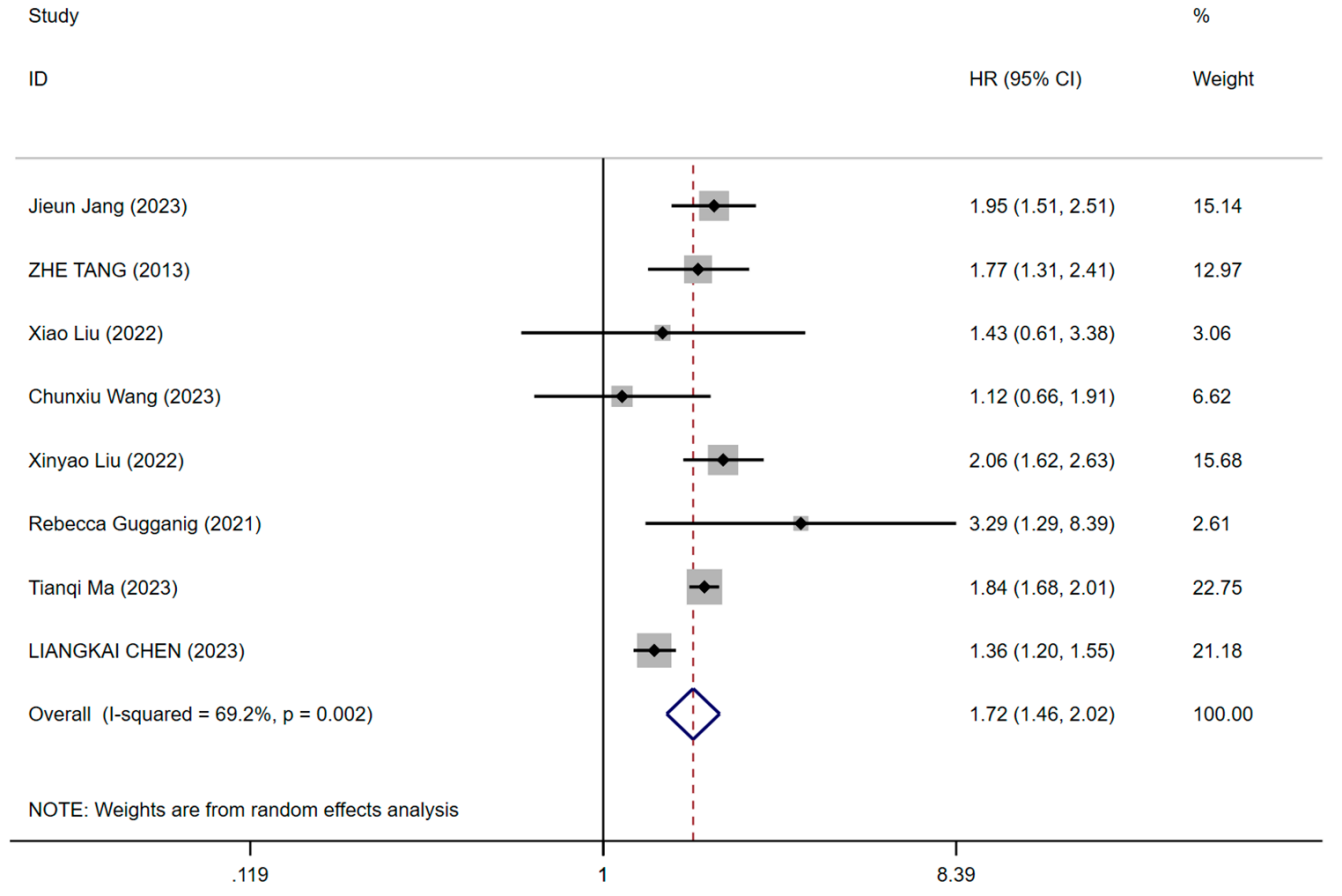
Forest plot from meta-analysis of stroke risk

Eight studies examined the relationship between pre-stroke frailty status and stroke risk [9, 17, 20, 37, 38, 40, 41, 43]. Two studies utilized FP to assess frailty, while the remaining six employed FI. Three employed retrospective cohort designs, whereas the remainder utilized prospective cohort designs. Six studies had a follow-up duration exceeding five years. The pooled RR of stroke for individuals with pre-stroke frailty compared to those without frailty was 1.72 (95% CI: 1.46–2.02, *p* = 0.002, *I*^*2*^ = 69.2%), as shown in Fig. 2. The pre-stroke frailty status was significantly associated with an increased risk of stroke with high significant heterogeneity between studies. The certainty of evidence was rated as low using the GRADE framework due to heterogeneity issues (*I²* = 69.2%, *p* = 0.002).

### Pre-stroke frailty and stroke mortality

**Fig. 3:**
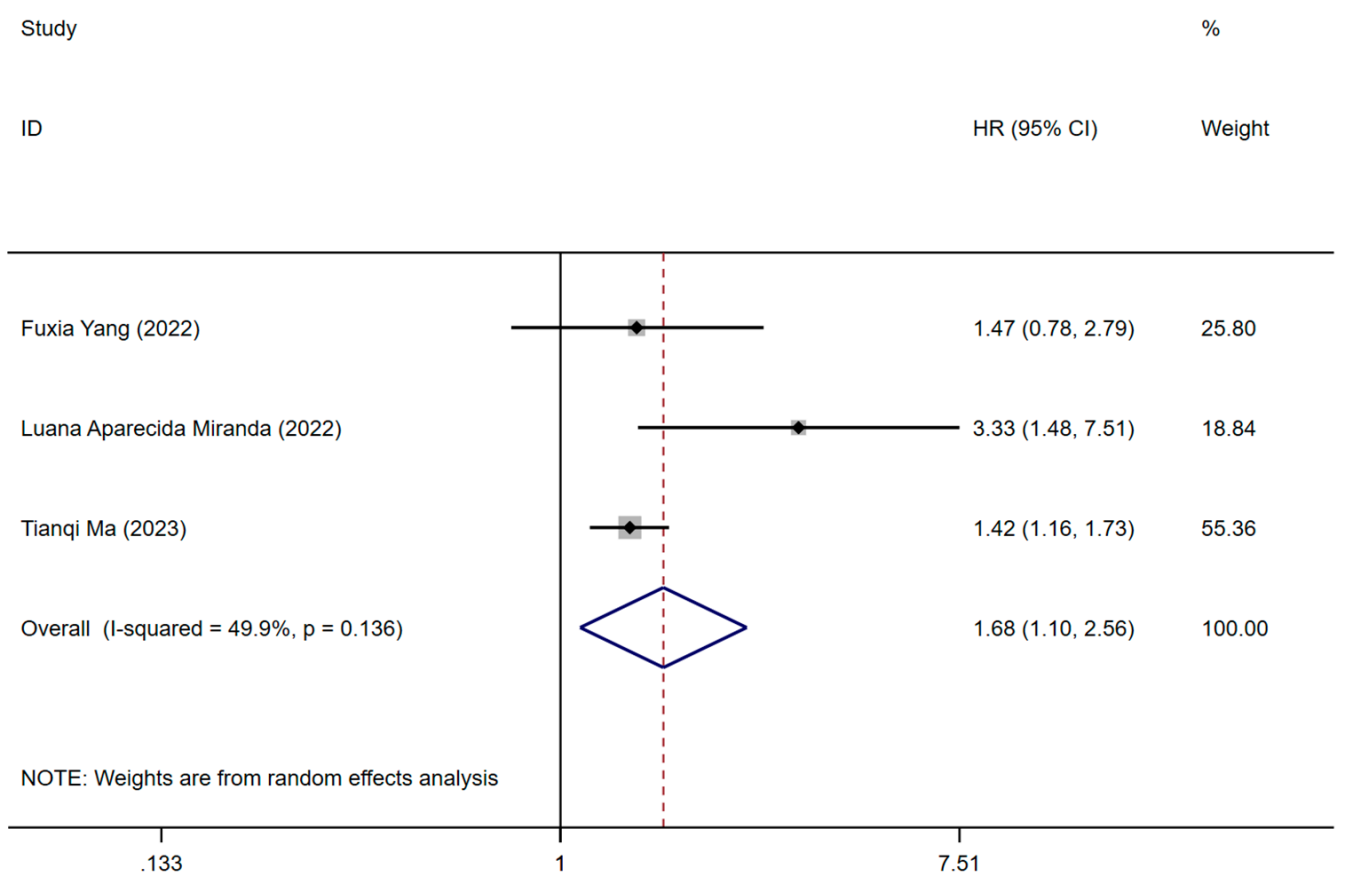
Forest plot from meta-analysis of stroke mortality

Three studies reported on the association of pre-stroke frailty status with stroke mortality [2, 38, 39]. One study assessed frailty utilizing FI, and the remaining two studies employed FRAIL and PRIMA-7. One employed retrospective cohort design, whereas the remainder utilized prospective cohort designs. All included studies used HR to measure stroke mortality, and the pooled HR for stroke mortality in pre-stroke frailty compared to non-frail individuals was 1.68 (95% CI: 1.10-2,56, *p* = 0.136, I^2^ = 49.9%), as shown in Fig. 3. Pre-stroke frailty was significantly associated with an increased mortality of stroke with low significant heterogeneity between studies. The certainty of evidence, assessed using the GRADE framework, was moderate.

### Pre-stroke frailty and mRs

**Fig. 4:**
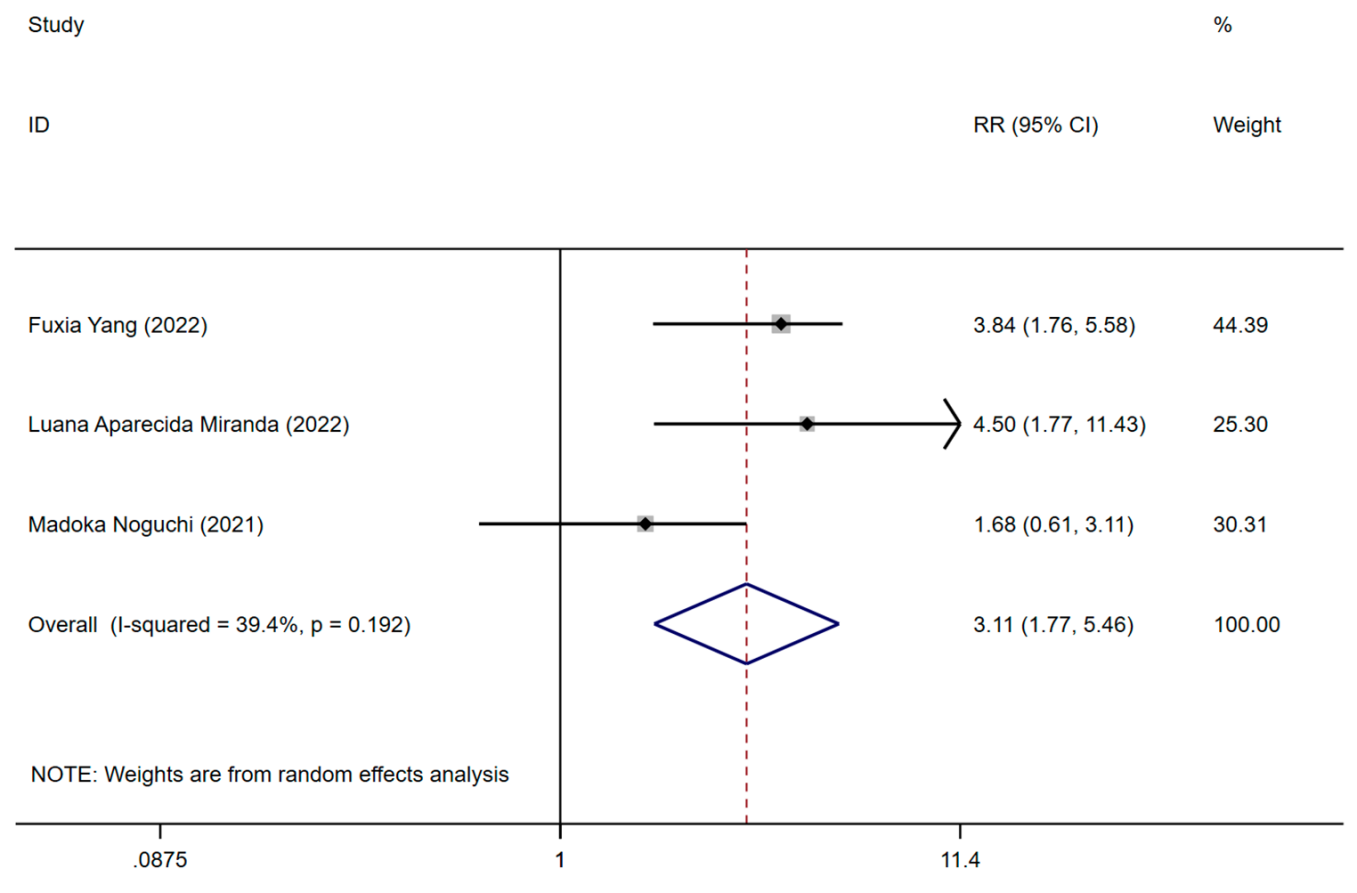
Forest plot from meta-analysis of mRs

Three studies reported on the association of pre-stroke frailty status with mRs [2, 39, 42]. The three studies respectively used FRAIL, PRIMA-7, and SFI to assess pre-stroke frailty. One employed retrospective cohort design, whereas the remainder utilized prospective cohort designs. To explore the RR, it was necessary to dichotomize the mRS scores into two groups, thereby converting them into binary variables. The studies in this meta-analysis used different methods to categorize mRS scores. Specifically, two studies classified mRS scores into two groups using a threshold of > 3, while another used a threshold of > 2. These groupings enabled consistent RR calculation across the studies in the analysis. One study reported mRS using RR, while the other two studies used OR. For the final analysis, OR values were converted to RR for consistency. The pooled RR for mRs for the presence of pre-stroke frailty compared to non-frail individuals was 3.11 (95% CI: 1.77–5.46, *p* = 0.192, *I*^*2*^ = 39.4%), as shown in Fig. 4. Pre-stroke frailty was significantly associated with an increased mRs of stroke with low significant heterogeneity between studies. The certainty of evidence, assessed using the GRADE framework, was moderate.

### Publication bias and sensitivity analysis

Begg’s rank correlation test indicated the absence of statistically significant evidence of publication bias across stroke risk (*p* = 0.536), mortality (*p* = 0.296), and mRS (*p* = 1.000). Furthermore, sensitivity analyses underscored the stability of the overall summary estimates, showing the summary estimates were not substantially influenced with exclusion of any one study (Figure S1-S3).

### Subgroup analysis

The results of the *I*^*2*^ analysis indicate that stroke risk had a high degree of heterogeneity. Therefore, we conducted subgroup analyses separately for the literature containing this outcome. Subgroup analyses stratified by follow-up period, frailty assessment tool, study type, and study quality revealed that the frailty assessment tool was the primary source of heterogeneity in the studies on the relationship between pre-stroke frailty and stroke risk. Subgroup analyses identified three sources of heterogeneity: frailty assessment tool, study type, and study quality. Figure S4 shows results from all subgroup analyses.

## Discussion

In an increasingly aging population, the effectiveness of traditional risk factors in estimating the risk of cardiovascular disease may diminish. Recent research has indicated that biological age may provide a more accurate prediction of adverse outcomes risk than chronological age. Actual age is not a reliable marker of physiological and functional status. The assessment of frailty serves as an emerging indicator of biological age [44]. There is growing recognition that identifying frailty aids in identifying risks of disease, adverse outcomes, and mortality [45]. Frailty, as a common geriatric syndrome, has been demonstrated to be closely associated with mortality, disability, healthcare expenditures, and unfavorable treatment outcomes. Moreover, evidence suggests that frailty is linked to an increased overall incidence of cardiovascular diseases compared to non-frail adults [39, 46–48]. Consequently, identifying individuals with frailty represents a public health priority [49]. Current evidence suggests that frailty may be reversible or delayed [14, 50], underscoring the significance of understanding the relationship between pre-stroke frailty and stroke risk for stroke prevention and management. This understanding not only aids healthcare professionals in better identifying and managing high-risk populations to implement appropriate preventive measures but also provides a reference for stroke intervention and treatment to enhance long-term prognosis. Although a substantial body of literature confirms the importance of frailty in cardiovascular disease [14, 51], this study represents the first investigation into the relationship between pre-stroke frailty, stroke risk, and its impact on post-stroke outcomes.

In the meta-analysis of pre-stroke frailty and stroke risk, we identified notable associations between pre-stroke frailty and stroke risk, as well as mortality and mRs. It is important to note that clinical factors, such as age, gender, smoking, drinking, hypertension, and diabetes, could confound the effects of pre-stroke frailty and stroke outcomes. In the studies we included, 10 studies accounted for controlling confounding factors to enhance the reliability of the outcomes. Pre-stroke frailty is frequently encountered and holds significant prognostic implications. It manifests in cognitive, social, psychological, oral, and nutritional domains [52]. There is a graded escalation in mortality risk corresponding to the accumulation of phenotypic components or deficits, the association has been corroborated across multiple studies and within various contexts and demographic subsets [47, 53, 54]. Population-based clinical studies have revealed that the prevalence of frailty among community-dwelling older adults ranges from 10–18% [37, 55], with approximately one-quarter of individuals aged 85 and older exhibiting frailty. The mechanism underlying frailty remain enigmatic. Despite several potential causes and intricate pathways being suggested, a comprehensive understanding of its etiology and pathogenesis remains elusive [56]. Frailty is currently considered to be associated with cellular aging, such as cells related to musculoskeletal and cerebral health [57]. Factors including genetics [58], gender [59], nutrition [60], oral health [61], and others are also closely linked to frailty. Therefore, intervening from multiple influencing factors to prevent frailty may be the most beneficial measure for reducing the incidence of frailty and mitigating its adverse effects.

The occurrence of frailty is not inevitable, instead, there is evidence indicating that health outcomes for individuals with frailty could be enhanced through multi-component interventions, preventive screening, and frailty assessment [62]. Based on this, in conjunction with our research findings, the risk of stroke and its adverse outcomes can be reduced through early screening and intervention for frailty. This suggests that systematic screening for frailty is crucial, particularly among elderly patients in the community. Studies demonstrate a negative correlation between frailty and quality of life. Additionally, frailty significantly diminishes patients’ self-care abilities, which will further impact the quality of life and disease risk among frail patients [63]. Highlighting the importance of early identification of frail individuals. It suggests targeted intervention measures should be implemented to prevent the onset of frailty, thereby enhancing the quality of life for patients [64, 65]. For instance, evidence suggests that adopting a dietary approach could serve as a practical method for mitigating “InflammAging” and for preventing or ameliorating frailty management, ultimately enhancing the care of elderly individuals [58]. In light of global aging trends, our focus on frailty should not only center on interventions for frail individuals, but should also incorporate primary healthcare principles into early frailty screening, thereby reducing the incidence of frailty for more efficient achievement of healthy aging. This initiative not only could diminish the disease burden on patients but also mitigates the societal healthcare burden.

In the meta-analysis examining the relationship between pre-stroke frailty status and stroke risk, significant heterogeneity was observed. We conducted subgroup analyses to explore its sources of heterogeneity, focusing on follow-up period, frailty assessment tool, study type, and study quality. In the subgroup analysis of pre-stroke frailty and stroke risk, the frailty assessment tool was identified as a source of heterogeneity. Since the seminal definition of frailty by Fried et al. [66]. , there has been a continuous proliferation of frailty assessment tools. In different studies, the optimal tool for screening and assessing frailty should be selected based on the characteristics of the subjects, the purpose of the assessment, and the clinical setting. As a result, researchers invariably employ a diverse array of assessment instruments. This, coupled with the lack of consensus regarding the definition and measurement of frailty, leading to confusion in both research and clinical contexts. The research indicates that the relationship between frailty and cerebral vessel disease remains debated, mainly due to the use of various frailty assessment methods [19]. Researchers also indicated that when comparing the capability of existing tools to identify frailty, only minimal to moderate levels of overlap are observed. Consequently, the inherent subtle flaws in this condition are easily overlooked, thereby impacting the prediction of adverse outcomes associated with frailty [44]. This implies that relying solely on a single frailty assessment tool for diagnosing frailty and guiding clinical decisions is unreliable. Therefore, the definition of frailty necessitates researchers to select the most suitable assessment tools based on the characteristics of the study population, aiming to mitigate biases arising from variations in assessment instruments. Although significant heterogeneity exists, regardless of the assessment method employed, there is a notable association between pre-stroke frailty occurrence and stroke risk, alongside its correlation with adverse outcomes. This bears implications for frailty screening initiatives and guiding post-stroke therapeutic and care pathways.

With the global population aging, the prevalence and impact of frailty are anticipated to grow. Regular frailty assessments for people, especially the elderly, are paramount. Conducting comprehensive frailty assessments for frail patients followed by personalized interventions can effectively alleviate the burden of frailty itself and significantly reduce the risk of stroke, thereby improving clinical outcomes. Indeed, recommendations from WHO also advocate for strengthening primary healthcare to provide preventive, multidisciplinary, and comprehensive management for elderly individuals, particularly those who are frail [67]. In this regard, there is a foreseeable need to identify populations requiring primary healthcare services for better management of frail patients. There are still unresolved inquiries awaiting future exploration, particularly regarding the most appropriate frailty assessment tools for diverse individuals. This entails considering both their prognostic accuracy and feasibility of use across different contexts. With the recognition of pre-stroke frailty’s significance in stroke, the subsequent query revolves around potential interventions. It’s imperative to ensure that amidst the push for a preventive strategy, public health services maintain the capacity to deliver comprehensive, multidisciplinary, and person-centered care, marking a crucial initial step.

### Advantages and limitations

Several notable advantages warrant mention. Firstly, this study represents the inaugural comprehensive exploration into the correlation between pre-stroke frailty and stroke risk, aligning squarely with the objectives of primary preventive care. Secondly, the utilization of a cohort design ensures the reliability of the data, as exposure data were procured prior to the onset of outcomes. Moreover, the inclusion of 1, 660, 328 independent studies with robust sample sizes confers strong statistical power for investigating these relationships. Nevertheless, it is imperative to acknowledge the limitations of this investigation. Firstly, the diverse frailty assessment methods used in the included studies led to diagnostic inconsistencies. Additionally, the lack of a standardized method for classifying mRS, despite performing heterogeneity and sensitivity analyses, may have introduced bias into the research findings. Secondly, while the multivariable adjusted model incorporated HR and RR, these results may have been affected by inherent or unmeasured confounding variables, including loss to follow-up and varying degrees of selection bias. Thirdly, Due to the limited literature on pre-stroke frailty and post-stroke activities of daily living (ADL), this study did not explore these related issues. Therefore, future research could focus on this aspect. Furthermore, this study’s literature search was limited to cohort studies in English and Chinese, excluding articles in other languages. This restriction is an additional constraint on the study’s generalizability.

## Conclusion

In conclusion, this study has demonstrated a significant association between pre-stroke frailty and an elevated risk of stroke, as well as adverse stroke outcomes. Our research contributed novel evidence supporting a causal relationship between pre-stroke frailty and stroke risk. Building upon this foundation, we underscore the significance of implementing frailty screening within community populations. Such initiatives greatly enhance primary preventive care efforts and yield a favorable impact on public health.

## Electronic supplementary material

Below is the link to the electronic supplementary material.

Supplementary Material 1

## Data Availability

No datasets were generated or analysed during the current study.
