# Supplementary material for: Association between pre-stroke frailty status and stroke risk and impact on outcomes: a systematic review and meta-analysis of 1,660,328 participants
Source: Aging Clin Exp Res. 2024 Sep 11;36(1):189. doi: 10.1007/s40520-024-02845-0 (PMC11390839; doi:10.1007/s40520-024-02845-0)
Supplement: Supplementary file 1 — Supplementary Material 1 [file 40520_2024_2845_MOESM1_ESM.docx]

**Association between pre-stroke frailty status and stroke risk and impact on outcomes: A systematic review and meta-analysis**

**Supplementary Table 1. Full Search Strategy**

| **PubMed** |
| --- |
| #1 "stroke"[MeSH Terms] OR "stroke"[Title/Abstract] OR "cerebrovascular accident*"[Title/Abstract] OR "cerebrovascular apoplexy"[Title/Abstract] OR "brain vascular accident*"[Title/Abstract] OR "cerebrovascular stroke*"[Title/Abstract] OR "apoplexy"[Title/Abstract] OR "cerebral stroke*"[Title/Abstract] OR "acute stroke*"[Title/Abstract] OR "acute cerebrovascular accident*"[Title/Abstract] |
| #2 "frailty"[MeSH Terms] OR "frailty syndrome"[Title/Abstract] OR "frailty"[Title/Abstract] |
| #3 "cohort stud*"[Title/Abstract] OR "longitudinal stud*"[Title/Abstract] |
| #4 #1 AND #2 AND #3 |
| **Web of science** |
| #1 TS=(stroke* OR "cerebrovascular accident*" OR "cerebrovascular apoplexy" OR "brain vascular accident*" OR "cerebrovascular stroke*" OR apoplexy OR "cerebral stroke*" "acute stroke*" OR "acute cerebrovascular accident*") |
| #2 TS=(frailty OR "frailty syndrome") |
| #3 TS=("cohort stud*" OR "longitudinal stud*") |
| #4 #1 AND #2 AND #3 |
| **Embase** |
| #1 (stroke* OR 'cerebrovascular accident*' OR 'cerebrovascular apoplexy' OR 'brain vascular accident*' OR 'cerebrovascular stroke*' OR apoplexy OR 'cerebral stroke*') AND 'acute stroke*' OR 'acute cerebrovascular accident* |
| #2 frailty OR 'frailty syndrome' |
| #3 'cohort stud*' OR 'longitudinal stud*' |
| #4 #1 AND #2 AND #3 |
| **Cochrane library** |
| #1. (stroke OR "cerebrovascular accident" OR "cerebrovascular apoplexy" OR "brain vascular accident" OR "cerebrovascular stroke" OR apoplexy OR "cerebral stroke" "acute stroke" OR "acute cerebrovascular accident"):ti,ab,kw |
| #2. ("cohort study" OR "longitudinal study"):ti,ab,kw |
| #3. (frailty OR "frailty syndrome"):ti,ab,kw |
| #4 #1 AND #2 AND #3 |
| **CINAHL** |
| #1. TI (stroke* OR 'cerebrovascular accident*' OR 'cerebrovascular apoplexy' OR 'brain vascular accident*' OR 'cerebrovascular stroke*' OR apoplexy OR 'cerebral stroke*' 'acute stroke*' OR 'acute cerebrovascular accident*') OR AB (stroke* OR 'cerebrovascular accident*' OR 'cerebrovascular apoplexy' OR 'brain vascular accident*' OR 'cerebrovascular stroke*' OR apoplexy OR 'cerebral stroke*' 'acute stroke*' OR 'acute cerebrovascular accident*') |
| #2. TI ('cohort stud*' OR 'longitudinal stud*') OR AB ('cohort stud*' OR 'longitudinal stud*') |
| #3. TI (frailty OR 'frailty syndrome') AND AB (frailty OR 'frailty syndrome') |
| #4 #1 AND #2 AND #3 |
| **PsycINFO** |
| #1. AB (stroke* OR 'cerebrovascular accident*' OR 'cerebrovascular apoplexy' OR 'brain vascular accident*' OR 'cerebrovascular stroke*' OR apoplexy OR 'cerebral stroke*' 'acute stroke*' OR 'acute cerebrovascular accident*') OR TI (stroke* OR 'cerebrovascular accident*' OR 'cerebrovascular apoplexy' OR 'brain vascular accident*' OR 'cerebrovascular stroke*' OR apoplexy OR 'cerebral stroke*' 'acute stroke*' OR 'acute cerebrovascular accident*') |
| #2. AB ('cohort stud*' OR 'longitudinal stud*') OR TI ('cohort stud*' OR 'longitudinal stud*') |
| #3. AB (frailty OR 'frailty syndrome') OR TI (frailty OR 'frailty syndrome') |
| #4 #1 AND #2 AND #3 |
| **CNKI** |
| ((((TS%= stroke or TI%= stroke) OR (TS%= cerebrovascular accident + cerebral infarction + cerebral hemorrhage)) AND ((TS%= frailty + frailty syndrome or TI%= frailty + frailty syndrome)) AND (TS% = cohort study))) |
| **Vip** |
| TS:(stroke or 'cerebrovascular accident' or 'cerebral infarction' or 'cerebral hemorrhage') and TS:(frailty or 'frailty syndrome') and TS:(cohort study) |

**Supplementary Table 2. List of** **articles excluded after full-text eligibility assessment**

| **Reasons for exclusion** | **Number** | **Reference** |
| --- | --- | --- |
| Lacking data on RR, HR or OR  Lack of original data hinders OR value conversion | 3  2 | [1-3]  [4, 5] |
| Not focus on the risk of the relationship between pre-stroke frailty and stroke and its outcomes | 5 | [6-10] |

**Reference:**

1. McEnhill, P., Torsney, K. M., & Rajkumar, C. Does frailty predict post stroke mortality? [J]. Age Ageing, 2020, 49(4): 535-536.

2. Damluji, A. A., Chung, S. E., Xue, Q. L., et al. Physical Frailty Phenotype and the Development of Geriatric Syndromes in Older Adults with Coronary Heart Disease [J]. Am J Med, 2021, 134(5): 662-671.e661.

3. Pilotto, A., Brass, C., Fassbender, K., et al. Premorbid frailty predicts short- and long-term outcomes of reperfusion treatment in acute stroke [J]. J Neurol, 2022, 269(6): 3338-3342.

4. Nozoe, M., Noguchi, M., Kubo, H., et al. Association between the coexistence of premorbid sarcopenia, frailty, and disability and functional outcome in older patients with acute stroke [J]. Geriatr Gerontol Int, 2022, 22(8): 642-647.

5. Joyce, N., Atkinson, T., Mc Guire, K., et al. Frailty and stroke thrombectomy outcomes-an observational cohort study [J]. Age Ageing, 2022, 51(2).

6. Zhu, X., Ding, L., Zhang, X., et al. Association of cognitive frailty and abdominal obesity with cardiometabolic multimorbidity among middle-aged and older adults: A longitudinal study [J]. J Affect Disord, 2023, 340: 523-528.

7. Zhang, Q., Gao, X., Huang, J., et al. Association of pre-stroke frailty and health-related factors with post-stroke functional independence among community-dwelling Chinese older adults [J]. J Stroke Cerebrovasc Dis, 2023, 32(6): 107130.

8. Tiainen, M., Martinez-Majander, N., Virtanen, P., et al. Clinical frailty and outcome after mechanical thrombectomy for stroke in patients aged ≥ 80 years [J]. J Stroke Cerebrovasc Dis, 2022, 31(12): 106816.

9. Winovich, D. T., Longstreth, W. T., Jr., Arnold, A. M., et al. Factors Associated With Ischemic Stroke Survival and Recovery in Older Adults [J]. Stroke, 2017, 48(7): 1818-1826.

10. Hanlon, P., Burton, J. K., Quinn, T. J., et al. Prevalence, measurement, and implications of frailty in stroke survivors: An analysis of three global aging cohorts [J]. Int J Stroke, 2023, 18(6): 720-727.

**Supplementary Table 3. Subgroup analysis of pre-stroke frailty associated with stroke risk**

| Study ID | Design | SELECTION | | | | COMPARABILITY | | OUTCOME | | | Grade |
| --- | --- | --- | --- | --- | --- | --- | --- | --- | --- | --- | --- |
|  |  | 1 | 2 | 3 | 4 | 1 | 2 | 1 | 2 | 3 |  |
| Jang 2023 | retrospective cohort | Yes | Yes | Yes | Yes | Yes | No | Yes | Yes | No | high |
| Ma 2023 | prospective cohort | Yes | Yes | Yes | Yes | Yes | No | Yes | Yes | No | high |
| Chen 2023 | prospective cohort | Yes | Yes | Yes | Yes | Yes | No | Yes | Yes | No | high |
| Wang 2023 | prospective cohort | Yes | Yes | Yes | Yes | Yes | No | Yes | No | Yes | high |
| Yang 2022 | prospective cohort | Yes | Yes | Yes | Yes | Yes | No | Yes | No | No | moderate |
| Miranda 2022 | prospective cohort | Yes | Yes | Yes | Yes | No | No | Yes | No | No | moderate |
| Xiao Liu 2022 | prospective cohort | Yes | Yes | Yes | Yes | Yes | No | Yes | Yes | No | high |
| Xinyao Liu 2022 | retrospective cohort | Yes | Yes | Yes | Yes | Yes | No | No | Yes | No | moderate |
| Gugganig 2021 | prospective cohort | Yes | Yes | Yes | Yes | Yes | No | Yes | No | Yes | high |
| Noguchi 2021 | prospective cohort | Yes | Yes | Yes | Yes | Yes | No | Yes | No | Yes | high |
| Tang 2013 | retrospective cohort | Yes | Yes | No | Yes | Yes | No | Yes | Yes | No | moderate |

**Supplementary Table 4. GRADE summary of findings table for the association between pre-stroke frailty and stroke risk/outcomes**

| **Certainty assessment** | | | | | | | **Effect** | | **Certainty** | **Importance** | |
| --- | --- | --- | --- | --- | --- | --- | --- | --- | --- | --- | --- |
| **№ of studies** | **Study design** | **Risk of bias** | **Inconsistency** | **Indirectness** | **Imprecision** | **Other considerations** | **Relative (95% CI)** | **Absolute (95% CI)** |  |  |  |
| **Risk of stroke** | | | | | | | | | | |  |
| 8 | non-randomised studies | not serious | serious | not serious | not serious | strong association | **HR 1.72** (1.46 to 2.02) | **2 fewer per 1,000** (from 2 fewer to 1 fewer) | ⨁⨁◯◯ Low | CRITICAL | |
| **Mortality** | | | | | | | | | | |  |
| 3 | non-randomised studies | not serious | not serious | not serious | not serious | strong association | **HR 1.68** (1.10 to 2.56) | **2 fewer per 1,000** (from 3 fewer to 1 fewer) | ⨁⨁⨁◯ Moderate | IMPORTANT | |
| **mRs** | | | | | | | | | | |  |
| 3 | non-randomised studies | not serious | not serious | not serious | not serious | strong association | **RR 3.11** (1.77 to 5.46) | **3 fewer per 1,000** (from 5 fewer to 2 fewer) | ⨁⨁⨁◯ Moderate | IMPORTANT | |

**CI:** confidence interval; **HR:** hazard ratio; **RR:** risk ratio

**
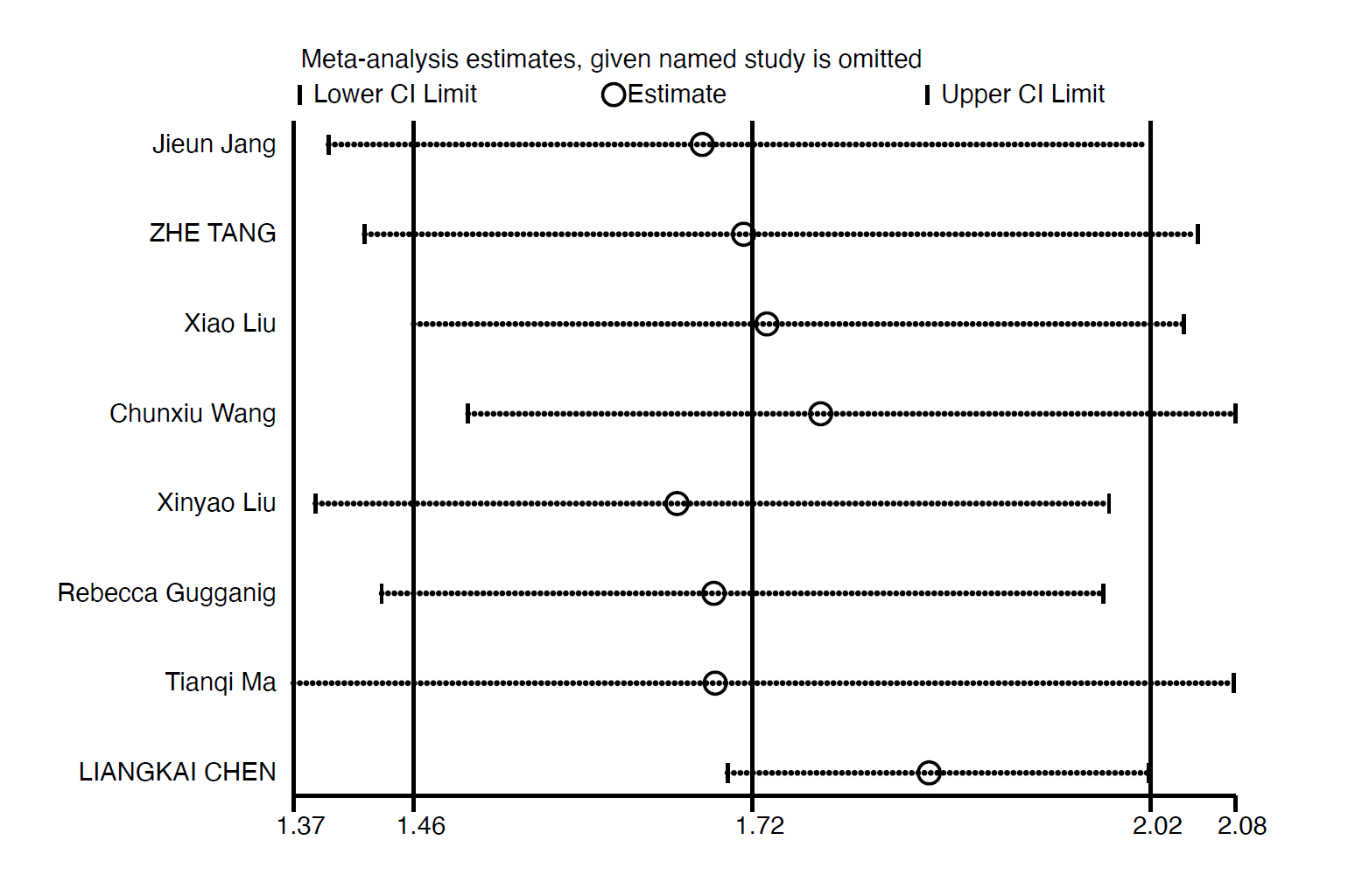
**

**Supplementary Figure 1. Sensitivity analysis results of stroke risk related literature.**

**
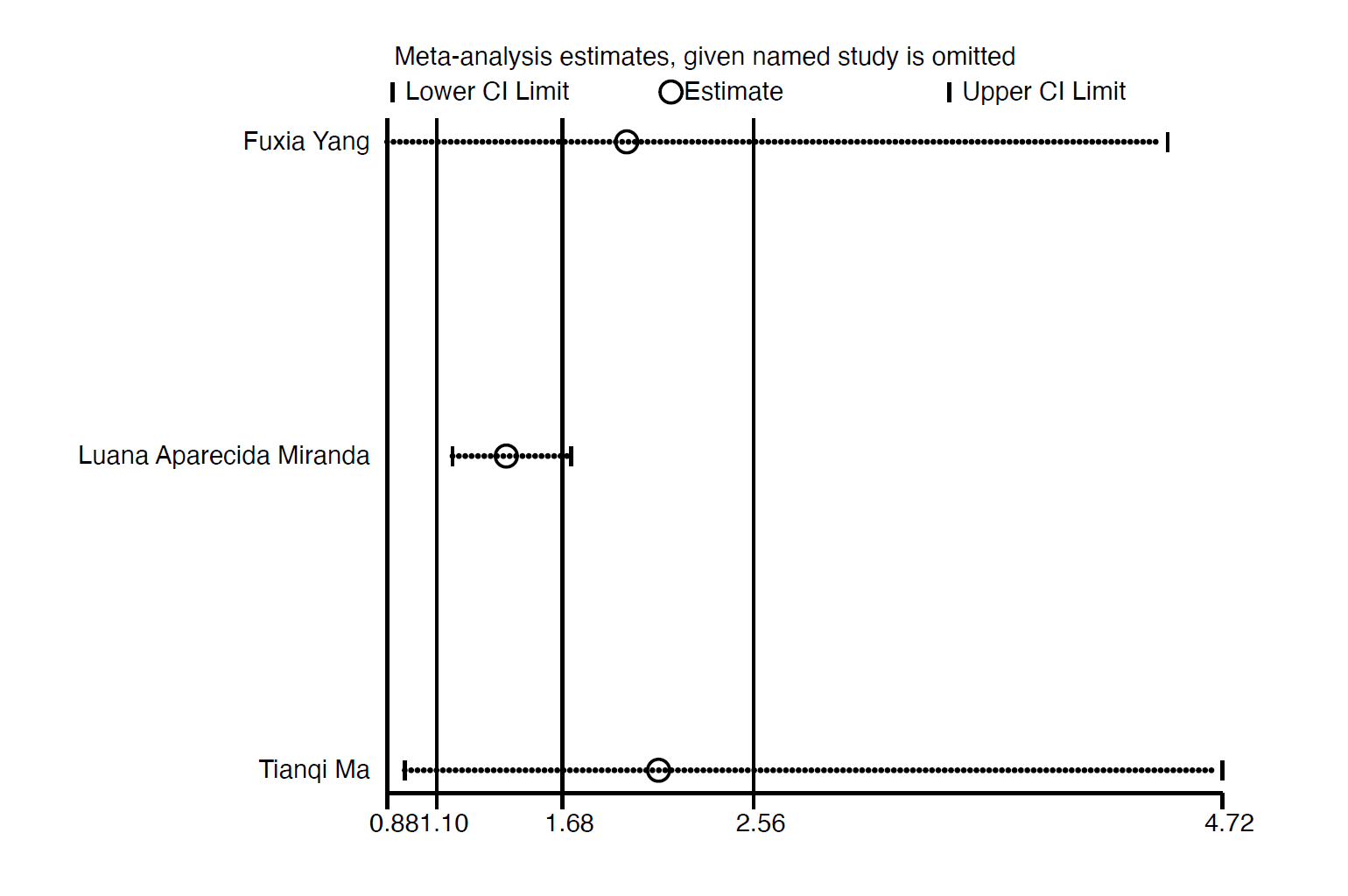
**

**Supplementary Figure 2. Sensitivity analysis results of mortality related literature.**

**
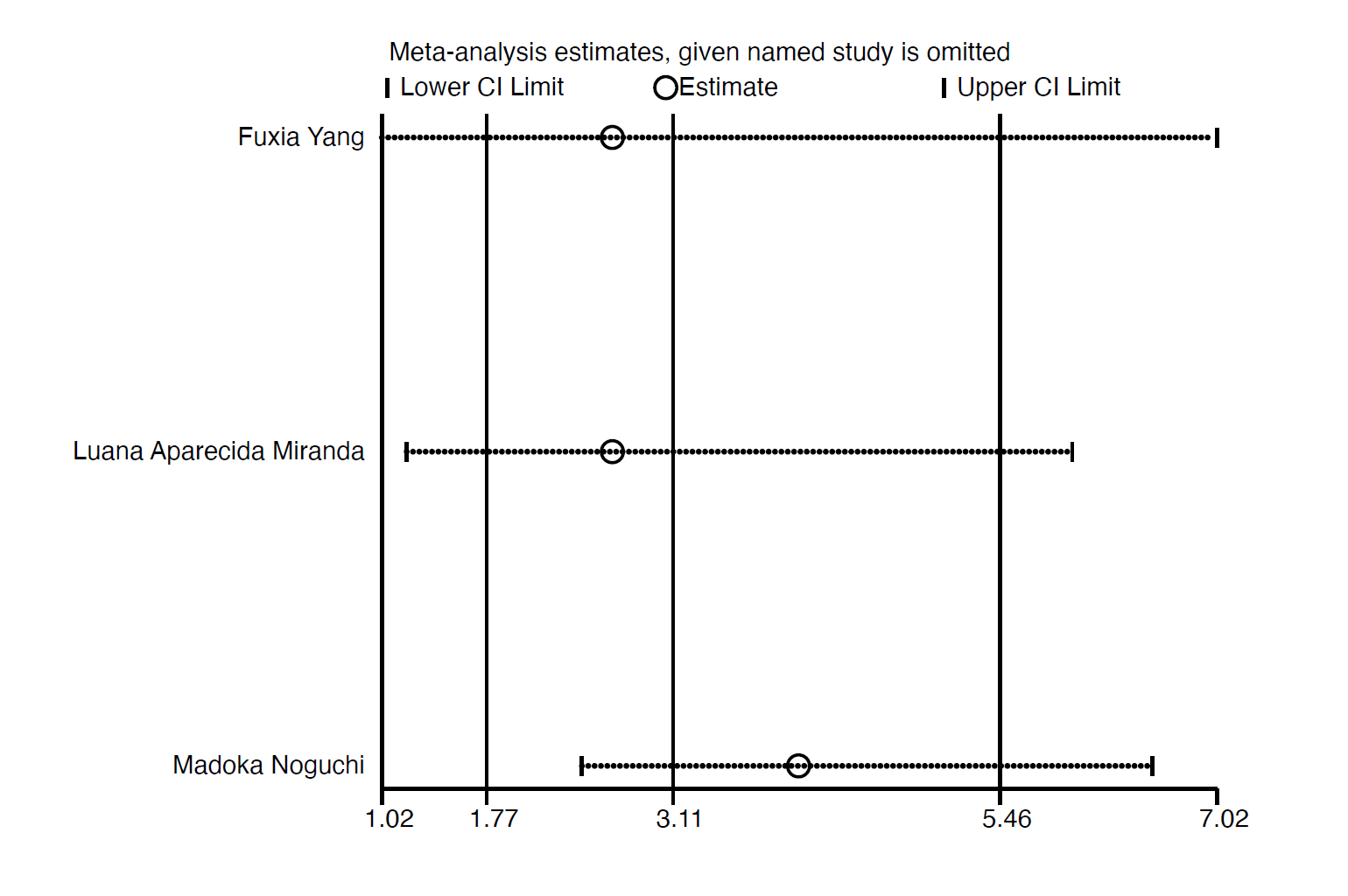
**

**Supplementary Figure 3. Sensitivity analysis results of mRs related literature.**

A.


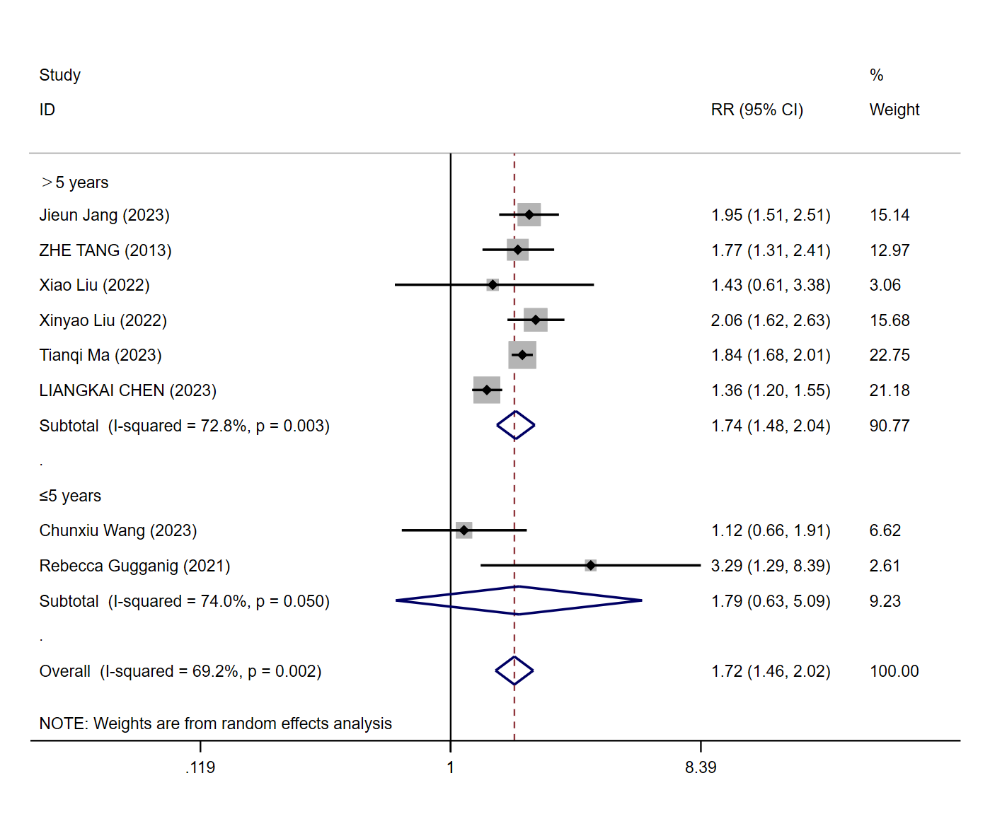


B.


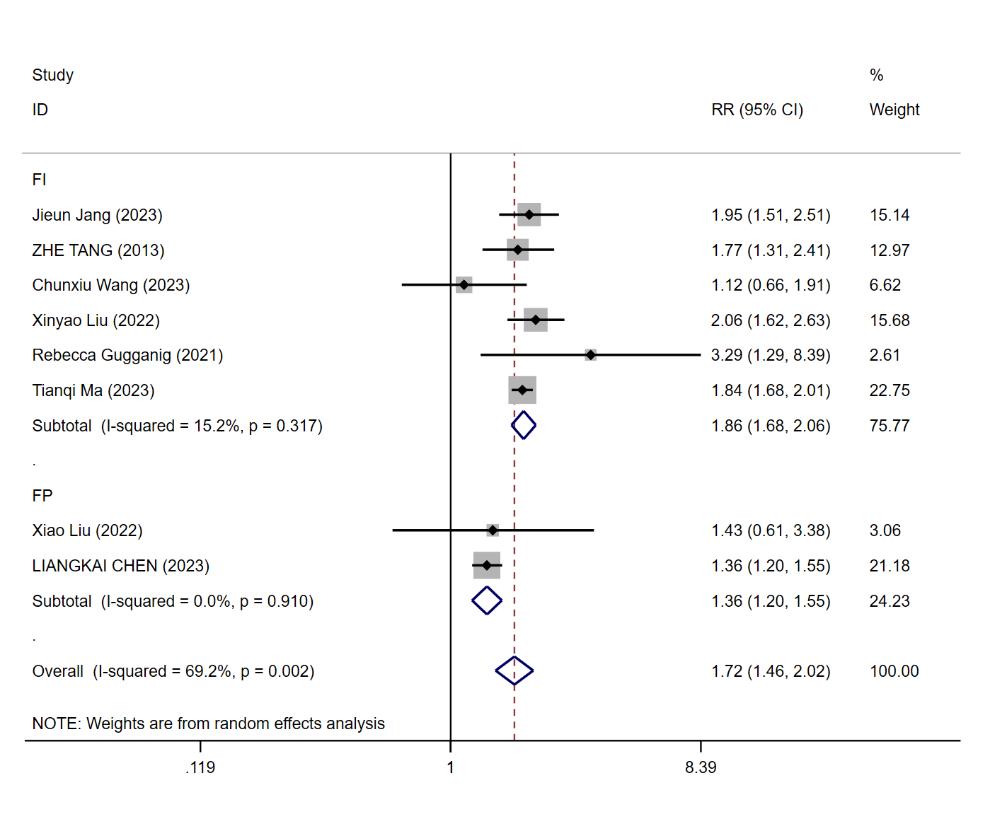


C.


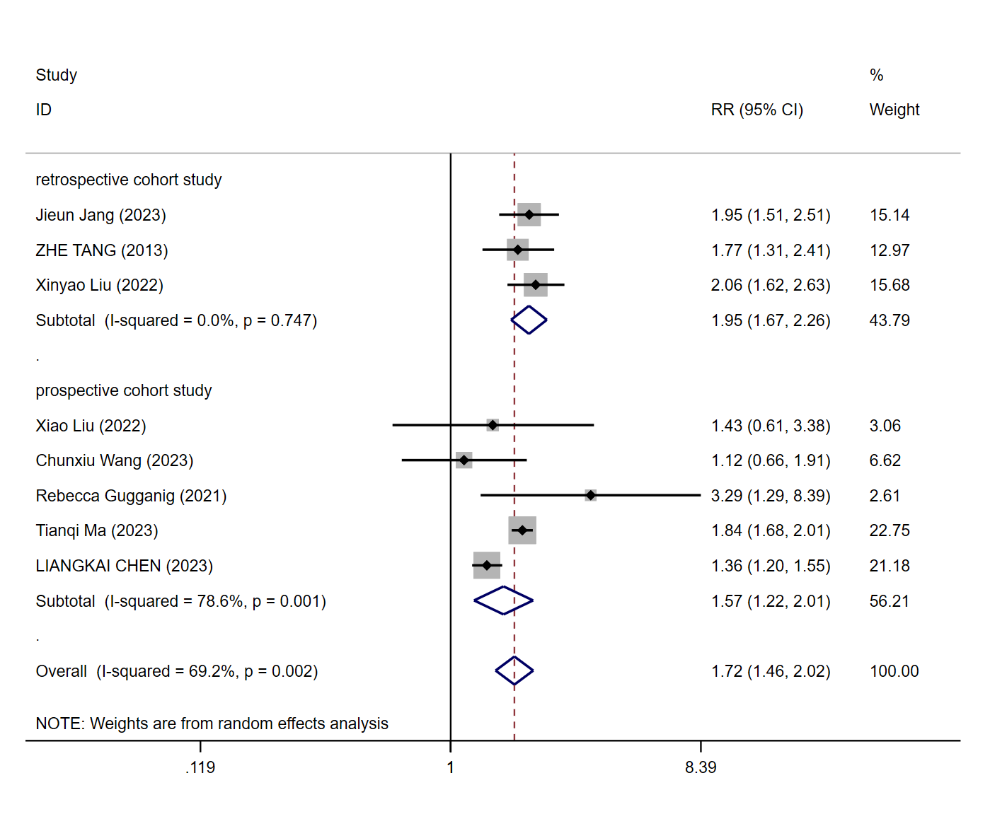


D.


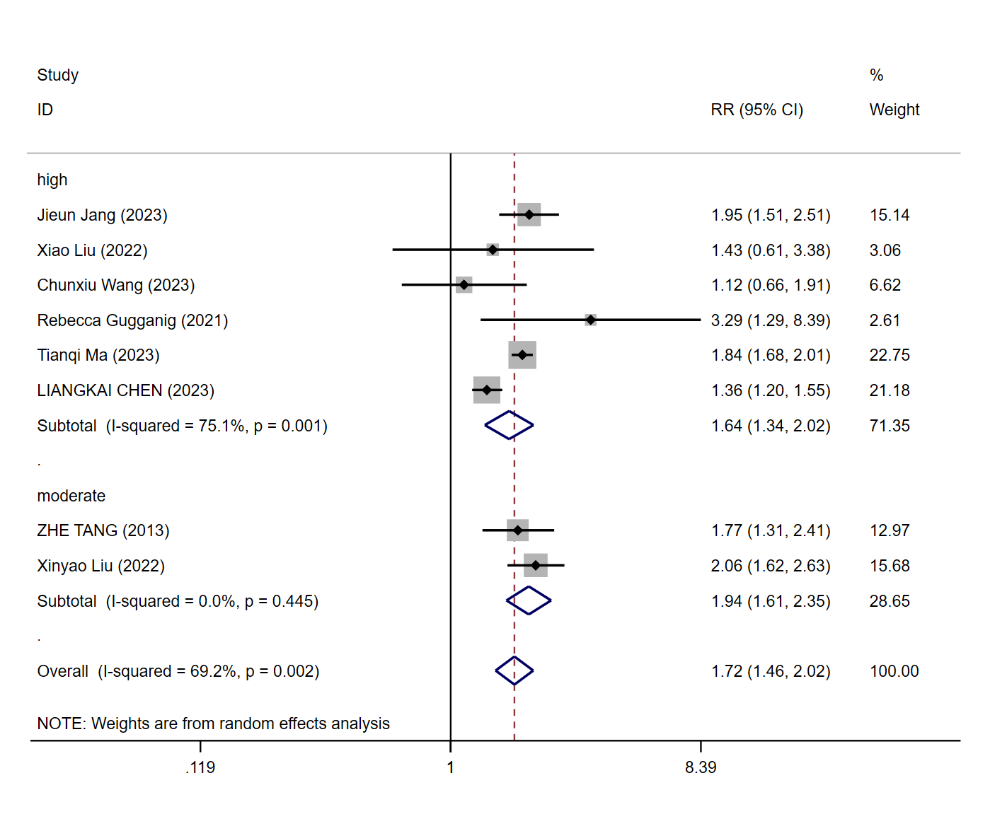


**Supplementary Figure 4. Subgroup analysis of pre-stroke frailty associated with stroke risk** (A: follow-up period; B: frailty assessment tool; C: study type; D: study quality)
